# Sex Differences and Emotion Regulation: An Event-Related Potential Study

**DOI:** 10.1371/journal.pone.0073475

**Published:** 2013-10-30

**Authors:** Elyse K. T. Gardener, Andrea R. Carr, Amy MacGregor, Kim L. Felmingham

**Affiliations:** School of Psychology, University of Tasmania, Hobart, Tasmania, Australia; The University of Queensland, Australia

## Abstract

Difficulties in emotion regulation have been implicated as a potential mechanism underlying anxiety and mood disorders. It is possible that sex differences in emotion regulation may contribute towards the heightened female prevalence for these disorders. Previous fMRI studies of sex differences in emotion regulation have shown mixed results, possibly due to difficulties in discriminating the component processes of early emotional reactivity and emotion regulation. The present study used event-related potentials (ERPs) to examine sex differences in N1 and N2 components (reflecting early emotional reactivity) and P3 and LPP components (reflecting emotion regulation). N1, N2, P3, and LPP were recorded from 20 men and 23 women who were instructed to “increase,” “decrease,” and “maintain” their emotional response during passive viewing of negative images. Results indicated that women had significantly greater N1 and N2 amplitudes (reflecting early emotional reactivity) to negative stimuli than men, supporting a female negativity bias. LPP amplitudes increased to the “increase” instruction, and women displayed greater LPP amplitudes than men to the “increase” instruction. There were no differences to the “decrease” instruction in women or men. These findings confirm predictions of the female negativity bias hypothesis and suggest that women have greater up-regulation of emotional responses to negative stimuli. This finding is highly significant in light of the female vulnerability for developing anxiety disorders.

## Introduction

There is an increasing interest in sex differences in emotion processing. This is particularly relevant for psychopathology as women have been shown to develop anxiety disorders and depression at twice the rate of men [1]. It has been proposed that sex differences in emotional reactivity may explain these different prevalence rates in men and women [2]. There is increasing recognition that difficulty regulating negative emotions is important in mediating anxiety and depression [3]. It is possible that greater difficulty regulating negative emotion may underlie the increased female prevalence for anxiety and depression. Very few studies have examined sex differences in emotion regulation processes. Emotion regulation in this study will be defined as the capacity to either down-regulate or up-regulate responses to emotional stimuli.

Event-related potential (ERP) studies examining sex differences to emotional stimuli suggest that women have greater reactivity in general than men. Campanella et al., (2004) [4] reported that whilst men and women displayed faster N2b latencies to fearful faces, women also displayed faster N2b latencies to happy faces. Proverbio et al., (2008) [5] found that women had greater N2 amplitude to negative stimuli from the International Affective Picture System (IAPS; [6] compared to men, particularly when stimuli depicted humans. Gasbarri et al. (2007) found increased P3 amplitude in the left hemisphere in women, and increased P3 amplitude in the right hemisphere in men in response to unpleasant IAPS images [7]. Han et al. (2008) found that painful stimuli elicited greater P3 amplitude compared to neutral stimuli for both sexes, and this effect was more pronounced in women [8]. This greater neural reactivity in women to emotional stimuli is consistent with proposals that there is greater emotional reactivity in women [9]. In particular, greater reactivity to negative stimuli may reflect a negativity bias in women, where women are more sensitized to threatening images than men [9].

In line with the female negativity bias, Li et al., (2008) reported that whilst both men and women showed increased N2 and P3 amplitude to highly negative images compared to neutral images, only women showed significantly greater N2 and P3 amplitude towards moderately negative images compared to neutral images [10]. They concluded that women have lower thresholds for responding to negative images than men. A recent study provides evidence that this female negativity bias occurs very early in processing emotional stimuli. Lithari et al., (2010) found greater N1 and N2 amplitudes to negative IAPS images in women and men, particularly in response to unpleasant images [11]. Lithari et al.(2010) concluded that women display greater early emotional reactivity to negative stimuli, consistent with a female negativity bias [11].

It is possible this female negativity bias in early automatic and cognitive processing may influence later emotion regulation. It is recognized that emotion regulation involves component processes of early emotional reactivity, as well as later conscious inhibitory control [12]. Some theorists suggest that an early negativity bias may deplete cognitive resources for later processing [9], whereas others suggest that an early negativity bias may enhance later processing and increase negative appraisals of negative stimuli, which may then impair the capacity to downregulate emotional responses [13].

The only studies investigating sex differences in emotion regulation are fMRI studies, and these report inconsistent results [14]. McRae, et al., (2008) recorded participants' brain activity during completion of an emotion regulation reappraisal task, which required participants to use a cognitive reappraisal strategy to “decrease” their emotional responses to negative emotional stimuli [15]. Men revealed reduced amygdala activity during emotion regulation to negative emotional stimuli compared to women, suggesting a greater capacity to regulate negative emotional responses. Concurrent with this decreased amygdala activity, men did not activate prefrontal networks involved in emotion regulation [16] to the same extent as women which was interpreted as reflecting more efficient emotion regulation processing in men [15]. In contrast, Domes et al, (2010) examined brain activity during a reappraisal task that instructed participants to “increase,” “decrease,” and “maintain” emotional responses to negative stimuli. Men were found to have greater activation in prefrontal regions than women, with no sex differences in amygdala activity, when regulating emotional responses to negative stimuli [17]. Domes et al. concluded that men may not have the more efficient emotion regulation processing system as claimed by McRae et al. (2008). This inconsistency may relate to the poor temporal resolution of fMRI, as it is difficult to discriminate the temporal dynamics between early emotional reactivity and later inhibitory emotion regulation.

Given their high temporal resolution, ERPs are a useful technique to discriminate early emotional reactivity from emotion regulation. N1 amplitude has been associated with increased engagement of autonomic arousal processes towards emotional stimuli [11], [18]. Increased N2 amplitude to negative emotional stimuli has been interpreted to reflect an increase in automatic processing of emotional facial expression [19], and emotional images [20]. Previous ERP research has reported an index of emotion regulation in the Late Positive Potential (LPP) waveform [21]. The LPP is characterised by a large positive slow-wave maximal at fronto-central midline sitesthat emerges between 400 and 800 ms following stimulus onset [21]. It has been reported as a consistent, non-habituating regulative response, which is highly sensitive towards high-arousing, emotionally salient stimuli compared to neutral stimuli [21].

The majority of ERP studies examining the LPP as a correlate of emotion regulation reveal that instructions to regulate emotional responses towards negative stimuli modulates LPP amplitude. That is, decreasing emotional responses through conscious modulation reduces LPP amplitude, while increasing emotional responses through conscious modulation increases LPP amplitude [13], [21]–[23].

The emotional ERP literature has revealed that the P3 is involved in the conscious appraisal of emotion, determining stimulus salience, and the modulation of emotional responses before later emotion regulation occurs [18], [22]. Bradley at al. (2003) suggested that within emotion regulation processing, the P3 component reflects “motivated attention” – the idea that emotion regulation processing directs, or motivates, attentional resources to facilitate later processing, due to the emotional stimuli's intrinsic motivational significance [24].

Previous research examining sex differences in emotion regulation have not discriminated between early emotional reactivity and later emotion regulation. The present study investigated sex differences in emotion regulation processing, specifically in early emotional reactivity (N1 and N2 components) and emotion regulation (P3 and LPP components) using ERP methodology. We hypothesized that women would display an early negativity bias towards negative stimuli, reflected in greater N1 and N2 amplitude to negative IAPS images. Further, we hypothesized that this sensitivity to negative stimuli would influence later emotion regulation processes reflected in LPP and P3. Specifically, we predicted that women would increase the LPP and P3 amplitude to an instruction to increase emotion to a greater extent than men, and would have less reduction in LPP and P3 amplitude to an instruction to decrease their responding to negative stimuli.

## Materials and Methods

### Participants

Forty-three first year psychology students (20 men, 23 women) were recruited to participate in the study in return for course credit. The mean age for men was 20.5 years ±0.6 and 20 years ±1.5 for women. All participants reported normal or corrected-to-normal vision. Potential participants were excluded if they were pregnant, heavy users of tobacco and alcohol, illicit drug and prescribed medication users, or had a history of mental illness, neurological disorders, head injuries, or any serious medical conditions. Participants were asked to refrain from alcohol, caffeine, and nicotine for three hours prior to the study to avoid confounds in ERP data. Written informed consent was obtained from adult participants in accordance with procedures of the Social Science Human Ethics Committee at the University of Tasmania. No minors were involved in this study. Participants completed the Depression Anxiety and Stress Scale (DASS: [25]) to assess mood state on the day of test, and the Emotion Regulation Scale [26] to assess emotion regulation style. This study was approved by the Social Sciences Human Ethics Committee at the University of Tasmania.

### Emotion regulation task

The emotion regulation task was adapted from the methodology of Moser et al. (2010: [13]). Each trial of the task consisted of an instruction word (“increase,” “decrease,” or “maintain”) was visually presented on the computer screen for 2000 ms, which instructed participants' in how to respond to an upcoming image. For “maintain” trials, participants were instructed to view the images and naturally respond to their content, with minimal or no alteration to the emotional responses the images evoked. For “increase” trials, participants were instructed to view the images as from an attached first-person perspective, as though the participants themselves or significant others were personally involved in the scenarios of the image (self-focused), or to imagine the image content and events as having a worsened outcome (situation-focused). The “increase” trials aimed to intensify the degree of emotion experienced. For “decrease” trials, participants were instructed to view the images as from a detached third-person perspective, specifically viewing the content of the images as fake, as if from a movie (self-focused), or to imagine the content of the images as having a positive outcome (situation-focused). The “decrease” trials aimed to reduce the degree of emotion experienced by the participant. The instruction word also acted as a fixation point in the centre of the screen to orient the participant's attention to the upcoming image. The image was presented immediately after the instruction word for 2000 ms, followed by a 3000 ms resting interval upon stimulus offset before the next trial commenced. The stimulus set for the passive viewing task consisted of 120 unpleasant, high-arousing colour images obtained from the IAPS [6] which were defined according to normative data. In line with Moser, no neutral images were presented (as it is not possible to regulate emotional reactions to neutral stimuli). Similar to Moser et al., the negative images had a mean normative arousal rating of 6.12, and a mean normative valence rating of 2.67. The selected images included scenarios involving human and animal mutilation, injury, or assaults. Women and men received the same negative and neutral IAPS images, in the same counterbalanced randomized order.

### Procedure

Participants had their skin cleaned and prepared for electrode attachment from a fitted electrode cap as per the International 10–20 system of electrode placement [27]. Participants were comfortably seated approximately 0.5 m from the STIM computer screen which displayed the emotion regulation task in a sound-proof room. Participants were instructed to maintain their eye gaze on the computer screen and to limit eye and body movement throughout all task trials.

The emotion regulation task was then thoroughly explained and participants completed 30 practice trials to familiarise themselves with the task. Two counterbalanced blocks consisting of 120 experimental trials each were then completed (participants viewed all IAPS images twice). One block randomly presented 60 “decrease” trials paired with 60 “maintain” trials (the “decrease” instructional set), while the other block randomly presented 60 “increase” trials paired with 60 “maintain” trials (the “increase” instructional set), following the procedure of Moser et al. (2010: [13]). Increase instructions were presented in a separate block from decrease instructions to prevent any contamination of emotion regulation strategies within each block. These blocks were presented in a randomized order, counterbalanced across the groups. For each trial, an IAPS image was displayed for 2000 ms, which was followed by a randomly occurring inter-trial interval ranging from 800 ms to1500 ms, and subsequently, a resting interval of 3000 ms prior to the commencement of the next trial. A block of 30 practice trials, separated into one “decrease/maintain” block (15 trials) and one “increase/maintain” block (15 trials), were completed before the experimental trials began. “Increase/maintain” and “decrease/maintain” trials did not appear simultaneously within blocks to avoid switching instructional sets within a block, which may have confounded data [28]. All instruction words and images within each block were counterbalanced and randomised across blocks, which were presented equally amongst women and men. Upon completion of the passive viewing task, a ten-point Likert-scale picture rating task was completed, which measured participant's subjective arousal and valence measures of the 120 IAPS images. Following the picture rating task, the post-task questionnaire was completed.

### ERP recording

Data were acquired using Neuroscan Stim^2^ software (Compumedics Neuroscan, 2003) on a Celeron D Class computer. EEG data were continuously recorded for the emotion regulation task using Neuroscan SCAN 4.5 software (Compumedics Neuroscan) on a SynAmps^2^ system connected to a 32 channel Quick-cap with silver (Ag) and silver chloride (AgCl) electrodes. Following previous emotion regulation ERP studies [13], EEG recordings were taken from five midline sites across the scalp based on inspection of the grand mean average data (Fz, FCz, Cz, CPz, Pz), as determined by the International 10–20 System of electrode placement [27], in accordance with Moser et al. (2010). All electrodes were referenced to linked mastoids, an AFz ground was used, and electro-oculogram (EOG) electrodes were positioned above and below the left eye as well as the outer canthi of each eye. Electrode impedances were maintained at or below 10 kΩ.

The continuous sampling rate for recorded data was 1000 Hz and amplified at 200 Hz. Data were rejected for vertical and horizontal EOG activity and artefacts exceeding +/−100 µV, were low-pass filtered at 30 Hz, and were epoched from 100 ms pre-stimulus to 1900 ms at stimulus offset. ERP components were selected relative to a 100 ms baseline (prior to each stimulus). Data were baseline corrected prior to selecting ERP components.

### Data analysis

Peak amplitudes of the N1 and N2 components were calculated by examining the peak (relative to baseline) ERP negative waveforms between 50–150 ms and 150–270 ms post-stimulus respectively, in accordance with previous research [13], [18], [20]. The P3 peak amplitude was calculated by examining the peak positive waveform at approximately 300 ms post-stimulus [21]. After inspection of grand mean average waveforms, the LPP amplitude activity was found to be maximal within the 400–800 ms post-stimulus onset time window at frontal regions. Mean LPP amplitude was then calculated for the specified time window.

N1, N2, P3, and LPP amplitude were analysed in separate 2 [Sex: Men, Women]×2 (Instruction: Increase/Maintain, or Decrease/Maintain)×3 (Site: Fz, FCz, Cz, or Cz, CPz, Pz) repeated measures analyses of variance (ANOVAs), with Sex as the between-subjects factor, and Instruction and Site as within-subjects factors. As per Moser et al. (2010), the present study separated the “increase” and “decrease” instructions for analysis (with separate respective baseline “maintain” comparison conditions) to prevent potential confounding of instructional sets. Following previous emotion ERP literature [11], [21], analyses of the N1, N2, and LPP amplitudes were focused on the fronto-central sites (Fz, FCz, Cz), while P3 amplitude data were analysed at the centro-parietal sites (Cz, CPz, Pz).

Significance levels were obtained at alpha <.05 with Greenhouse-Geisser corrections employed when there was significant sphericity in the repeated measures data. Effect sizes were calculated and measured using partial eta squared (ηp^2^), and 95% confidence intervals were obtained. Significant interactions and main effects were further analysed using Sidak-adjusted pairwise comparisons, and Hedge's *g*, a *d*-type effect size statistic, was used to estimate effect sizes for Sidak-adjusted pairwise comparisons. Data were analysed using the Statistical Package for the Social Sciences (SPSS) version nineteen.

## Results

### Demographic and clinical data

Separate univariate ANOVAs with Sex as the between-subjects factor were conducted to determine if there were any group differences in age, self-reported depressed mood, anxiety, and stress (as measured by the DASS; [25]), and self-reported reappraisal and suppression emotion regulation strategies (as measured by the ERS; [26]). Table 1 presents a summary of these analyses. No significant differences were found between men and women in age, depressed mood, anxiety, stress, or emotion regulation strategies.

**Table 1:** Mean Scores for Age, Depressed Mood, Anxiety, Stress, and Reappraisal and Suppression Emotion Regulation Strategies for Men and Women (Standard Deviations in Parentheses).

Variable	Men	Women	F	P	η_p_^2^
Age	20.5(2.6)	20.0(3.5)	.32	.57	.01
Depressed Mood	4.7(5.9)	4.9(5.3)	.01	.92	.001
Anxiety	4.5(5.6)	3.1(3.6)	.95	.34	.02
Stress	8.3(7.3)	11.5(6.2)	2.39	.13	.06
Reappraisal	29.3(5.21)	28.3(4.22)	.48	.49	.01
Suppression	15.9(5.44)	14.6(4.25)	.82	.37	.02

### ERP data

**Figure 1:**
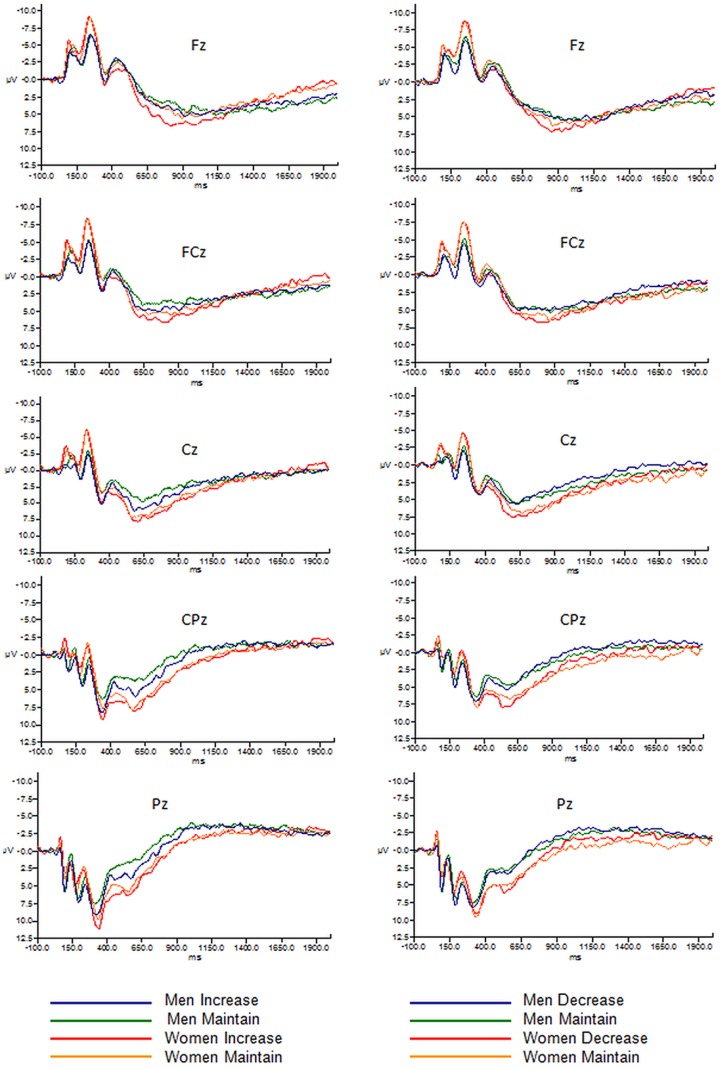
Grand mean average waveforms, including amplitude (µV) from midline regions for the “increase” and “decrease” instructional set for men and women.

There were no significant differences between groups in the number of rejected trials. Grand mean average waveforms for men and women across each instruction group are depicted in Figure 1. The N1 (Site Increase/Maintain: *F* (5,205) = 3.1, *p* = .01; Site Decrease/Maintain: *F* (5,205) = 10.7, *p* = .000), N2 (Site: Increase/Maintain: *F* (5,205) = 49.9, *p* = .010; Site Decrease/Maintain: *F* (5,205) = 53.7, *p* = .000) and LPP components (Site: Increase/Maintain: *F* (5, 205) = 68.6, *p* = .000; Site Decrease/Maintain: *F* (5,205) = 105.7, *p* = .000) were maximal at the frontal, fronto-central, and central midline region (Fz, FCz, Cz) which accords with previous literature [11], [13]. The P3 component (Site Decrease/Maintain: *F* (5,205) = 57.8, *p* = .000; Site Increase/Maintain: *F* (5,205) = 54.9, *p* = .000) was maximal at the central, centro-parietal, and parietal midline sites (Cz, CPz, Pz), consistent with previous literature [21]. The LPP component had a maximal amplitude activity in the 400–800 ms time window, similar to Hajcak et al [21]. Following previous studies, analyses were restricted to sites in which ERP components were maximal.

### Increase vs maintain instructions

#### N1

**Figure 2:**
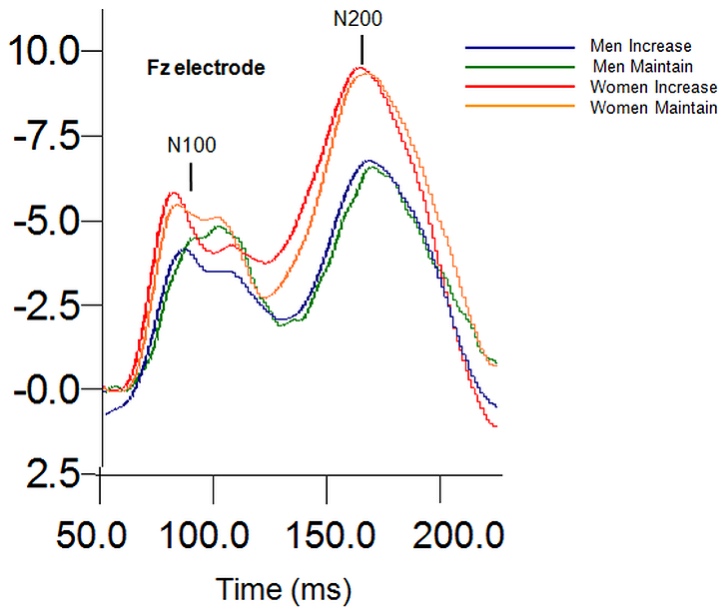
Sex main effects at N100 and N200 amplitude to the “increase” instructional set.

For N1 amplitude, a significant main effect of Sex was found, *F* (1, 41) = 9.23, *p* = .004, η_p_^2^ = .184 (see Figure 2). Women showed significantly greater N1 amplitude compared to men. No other main effects or significant interactions were found.

#### N2

A significant main effect of Sex was found for N2 amplitude, *F* (1, 41) = 6.59, *p* = .014, η_p_^2^ = .138, where women showed greater N2 amplitudes compared to men (see Figure 2).

#### P3

**Figure 3:**
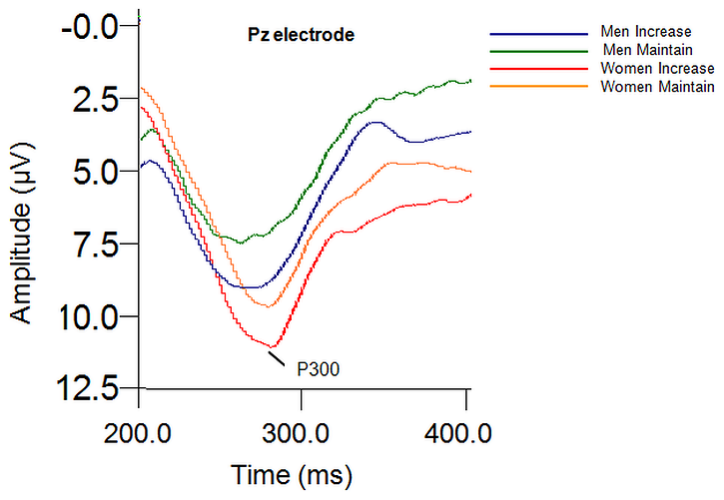
Instruction main effect at P300 amplitude to the “increase” instructional set.

For P3 amplitude, there was a significant main effect of Instruction, *F* (1, 82) = 29.755, *p* = .0001, η_p_^2^ = .421, revealing greater P3 amplitude to the “increase” instruction compared to the “maintain” instruction (see Figure 3). No other significant main effects or interactions were found for P3 amplitude.

#### LPP

**Figure 4:**
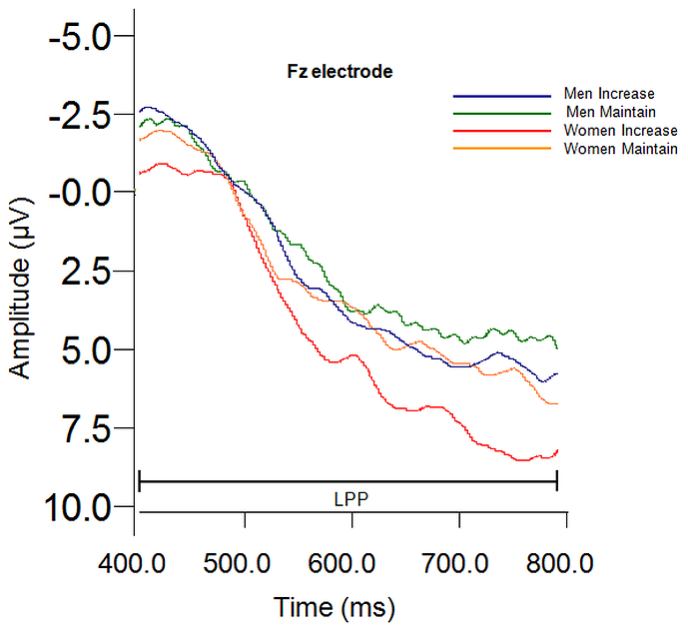
Instruction×Site×Sex interaction for mean LPP amplitude to the “increase” instructional set.

Significant main effects of Sex, *F* (1, 41) = 7.16, *p* = .011, η_p_^2^ = .149, Instruction, *F* (1, 82) = 10.44, *p* = .002, η_p_^2^ = .203, and Site×Sex, *F* (1.58, 64.59) = 4.44, *p* = .023, η_p_^2^ = .098, Greenhouse-Geisser corrected, ε = .788, were found for LPP amplitude. These significant main effects and interactions were subsumed by a significant Instruction×Site×Sex interaction, *F* (1.85, 75.91) = 5.271, *p* = .009, η_p_^2^ = .114, Greenhouse-Geisser corrected, ε = .926. Separate two-way Sex [Men, Women]×Instruction (Increase, Maintain) repeated measures ANOVAs with Sidak-adjusted pairwise comparisons were conducted at each fronto-central site (Fz, FCz, Cz) to identify where significant effects lay in this three-way interaction. These breakdown analyses revealed that men and women differed significantly in LPP amplitude between the “increase” and “maintain” instructions at Fz, *F* (1, 41) = 8.024, *p* = .007, η_p_^2^ = .164 (see Figure 4), but there were no sex differences at Fcz or Cz.

**Figure 5:**
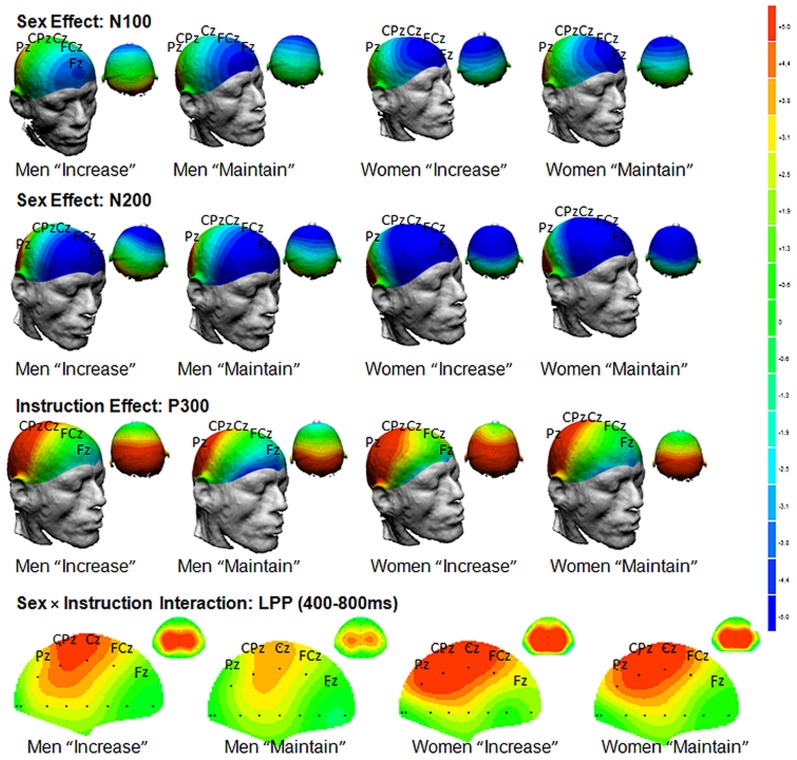
Topographies of the Sex main effects at N100 and N200 amplitude, the Instruction main effect at P300 amplitude, and the Instruction×Site×Sex interaction for mean LPP amplitude to the ‘increase” instructional set (blue negative, red positive).

Sidak-adjusted pairwise comparisons revealed that at Fz, women showed significantly enhanced LPP amplitudes to the “increase” instruction compared to the “maintain” instruction, but men did not reveal increased LPP amplitudes to “increase” versus “maintain” instructions. A summary of findings and topographic distribution of effects for “increase” versus “maintain” conditions is presented in Figure 5.

### Backwards stepwise multiple regression analyses

To examine the relative effect of sex on ERP amplitudes, we conducted four stepwise multiple regressions with N1, N2, P3 and LPP amplitude from the increase-maintain condition as dependent variables, and depressed mood, anxious mood, stressed mood, reappraisal, suppression, age and sex as predictor variables entered in the same order as predictors (see tables in supporting information). N1 amplitude (Table S1) was significantly predicted by Sex (Beta = .37, *t* = 2.7, *p* = .010) and age (Beta = .28, *t* = 2.04, *p* = .048), N2 amplitude (Table S2) was significantly predicted by Sex (Beta = .43, *t* = 2.98, *p* = .005) and stress (Beta = .15, *t* = 2.2, *p* = .035), P3 amplitude (Table S3) was predicted by Sex (Beta = −.35, t = −2.4, p = .023), and LPP amplitude (Table S4) was predicted by Sex (Beta = −.36, *t* = −2.5, *p* = .017).

### Decrease vs maintain instructions

#### N1

**Figure 6:**
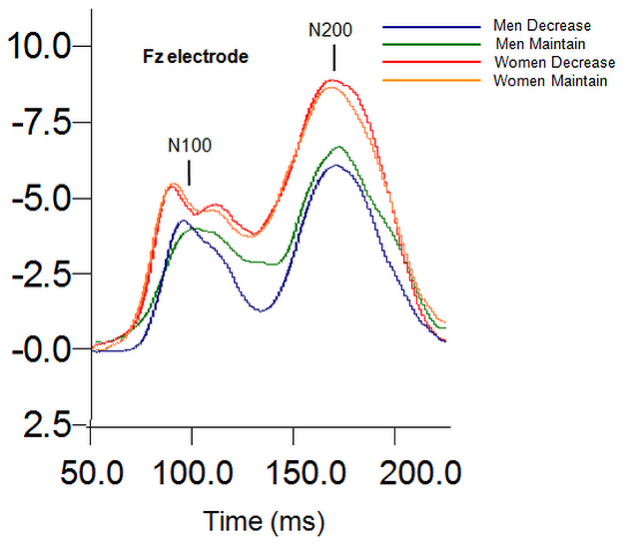
The Site×Sex interaction for mean N100 amplitude to the “decrease” instructional set.

For N1 amplitude, a significant main effect of Sex was found, *F* (1, 41) = 20.41, *p* = .0001, η_p_^2^ = .332, however this main effect was subsumed by a significant Sex×Site interaction, *F* (1.5, 60.6) = 10.164, *p* = .001, η_p_^2^ = .199, Greenhouse-Geisser corrected, ε = .739 (see Figure 6).

Sidak-adjusted pairwise comparisons revealed that women had significantly greater N1 amplitudes compared to men at Fz, *p* = .0001, Hedge's *g* = 1.45, at FCz, *p* = .0001, Hedge's *g* = 1.42, and at Cz, *p* = .003, Hedge's *g* = .986. No significant main effects or interactions were found for N2, P3 or LPP amplitude in the decrease-maintain condition.

No significant main effects or interactions for N2 amplitude, P3 amplitude or LPP amplitude were found in the decrease compared to maintain condition.

### Picture rating task and post-task questionnaire data

Two separate univariate ANOVAs with Sex as the between-subjects factor were conducted to determine if there were any significant sex differences in the picture rating task data. No significant sex differences were found between arousal and valence ratings of the images include in the decrease/maintain or increase/maintain blocks (see Table 2).

**Table 2:** Mean Scores for Arousal, Valence, Emotional Intensity (EI), and Utilised Effort for Instructions for Men and Women (Standard Deviations in Parentheses).

Variable	Men	Women	F	P	η_p_^2^
Arousal	3.59(1.6)	3.86(1.8)	.27	.61	.006
Valence	3.95(0.65)	3.72(0.65)	1.33	.26	.031
EI (Maintain)	3.25(1.45)	3.57(1.5)	.48	.49	.012
EI (Increase)	5.15(1.42)	5.00(1.41)	.12	.73	.003
EI(Decrease)	2.15(1.31)	3.57(1.67)	9.33	.004	.185
Effort(Maintain)	2.70(1.66)	3.83(2.02)	3.93	.054	.087
Effort(Increase)	5.35(1.27)	5.00(1.35)	.76	.39	.018
Effort(Decrease)	3.90(1.45)	4.70(1.82)	2.46	.12	.06

Separate univariate ANOVAs with Sex as the between-subjects factor were performed to determine if there were any sex differences in the post-task questionnaire data (see Table 2). There were no significant sex differences in perceived effort required to utilise the “increase,” “decrease,” and “maintain” instructions (all *p* values >.05). There were also no sex differences in the emotional intensity experienced to the image following the “increase” and “maintain” instructions (both *p* values >.05), however there was a significant sex difference in the emotional intensity experienced while following the “decrease” instruction (*p* = .004). Women reported greater emotional intensity to negative images following the “decrease” instruction compared to men.

## Discussion

This study examined sex differences in emotion regulation processing, specifically in terms of early emotional reactivity and later emotion regulation processing. The major findings were that women had enhanced N1 and N2 amplitudes compared to men to negative IAPS images, reflecting greater early emotional reactivity in women. In terms of emotion regulation, there were greater P3 and LPP amplitudes to “increase” instructions compared to “maintain” instructions, but no changes in P3 and LPP to “decrease” instructions. Furthermore, women displayed significantly greater P3 and LPP amplitudes to the “increase” instruction compared to the “maintain” instruction than men, who did not reveal increased P3 and LPP amplitude to the “increase” instruction. No sex differences were observed to the “decrease” emotion instruction.

### Sex differences in early emotional reactivity: N1 and N2

Main effects of sex showed that women had significantly greater N1 and N2 amplitude to negative stimuli (in the increase-maintain condition for N2) compared to men. These findings replicate a previous ERP emotion study, as Lithari et al. (2010) found that women showed greater N1 and N2 amplitudes to high-arousing negative images compared to men [11]. Lithari et al. (2010) interpreted this finding to reflect greater early emotional reactivity in women than men. This interpretation of greater emotional reactivity in women is consistent with fMRI findings of greater limbic and amygdala activity in response to negative stimuli in women compared to men [29], [30]. Taken together, these findings suggest that women have greater early emotional reactivity to negative stimuli, thus supporting a female negativity bias. The lack of significant findings for N2 amplitude to the “decrease” instructional set may potentially be due to overall lower responding in both men and women.

### The effect of the emotion regulation instruction: P3 and LPP

Findings revealed that P3 and LPP amplitudes were significantly enhanced to “increase” compared to “maintain” instructions. This finding replicates previous ERP literature that has used similar emotion regulation instructions [13], [22]. The increase in LPP amplitude was found predominantly at the frontal site (Fz), which is consistent with previous findings [13]. Although the spatial resolution of ERPs is limited, this frontal topography may reflect a general frontal cortical activation in response to “increase” emotion instructions. This topographic distribution is also broadly consistent with previous fMRI research in emotion regulation, where emotion regulation instructions predominantly influence prefrontal cortical regions [16].

While P3 has not been explicitly studied in response to emotion regulation instructions, as it reflects the conscious appraisal of emotion, it may influence emotion regulation through prioritising the allocation of attention resources to emotional stimuli over neutral stimuli [21]. Previous research has revealed increases in P3 amplitude towards negative emotional stimuli compared to neutral stimuli [31], [32]. As the present study found that the “increase” instruction resulted in greater P3 amplitudes over the “maintain” instruction, this may reflect a greater allocation of attention resources to negative stimuli in response to the “increase” emotion instruction.

In contrast, for the “decrease” instructional set, P3 and LPP amplitudes did not significantly differ between the “decrease” instruction and the “maintain” instruction. This result is consistent with Moser et al. (2010) as they did not find a reduction in LPP amplitude at 400–800 ms after a similar “decrease’ instruction [13]. However, Moser et al. did find a significant reduction in LPP amplitude for the “decrease” instruction at a much later time-window (1000–1800 ms) and it occurred only at the parietal midline site. This later decrease in LPP may reflect a suppressive than reappraisal emotion regulation strategy. Specifically, this later timeframe in Moser et al.'s study may reflect expressive suppression of the emotional response, which requires the emotional response to occur before suppression is instigated. Other emotion regulation studies have also reported a reduction in LPP amplitude to a “decrease” emotion instruction [33], [34]. This may have resulted from the use of different words in the instructions as previous studies have used the term “suppress” rather than an instructional set which encourages reappraisal of the stimulus.

### Sex differences in emotion regulation: P3 and LPP

Women showed significantly enhanced LPP amplitude when following “increase” instructions compared to the “maintain” instructions relative to men at Fz, who did not differ in LPP amplitude between the “increase” and “maintain” instructions. There were no significant interactions of sex and instructional set on P3 amplitude. The present study's LPP amplitude results suggest that women engaged greater emotion regulation resources in response to instructions to “increase” emotional responses than men. This finding may relate enhanced N1 and N2 amplitude to negative stimuli observed in women [11]. That is, greater early emotional reactivity to negative stimuli may have led to greater cognitive resources being made available in women, supporting a female negativity bias that extends from automatic (N1, N2) to conscious cognitive processes (LPP). This is consistent with Moser et al.'s (2010) interpretation that an increase in LPP amplitude to “increase” emotion instructions may involve both arousal and the recruitment of additional cognitive resources [13]. Our finding that the LPP amplitude at fronto-central sites was elevated to “increase” instructions in women are in line with previous fMRI studies where women have shown enhanced activity and greater engagement of limbic arousal and particularly prefrontal regions to increasing emotional responses to negative stimuli [15].

The final hypothesis predicting that women would show less reduction of P3 and LPP amplitudes to the “decrease” instruction than men was not supported. The P3 and LPP amplitude results revealed that neither men nor women showed significant reductions in P3 and LPP amplitudes to the “decrease” instruction compared to the “maintain” instruction. These results are not consistent with previous fMRI research that found significant reductions in limbic and prefrontal regions when consciously decreasing emotional responses towards negative emotional stimuli particularly for men and, to a significantly lesser extent, women [15]. It is likely that these null sex differences are due to the lack of effect of the “decrease” instructional set. Based on this information, it would be hasty to draw conclusions about sex differences in response to the “decrease” instructions, and future research should explore ways to enhance the efficacy of the “decrease” instruction.

The increased LPP amplitude to the “increase” instruction in women may reflect greater emotional appraisal towards negative emotional stimuli, confirming predictions of Moser et al., (2010) that early emotional reactivity can enhance subsequent negative appraisals [13]. Based on evolutionary pressures, it is possible that women are simply more biologically sensitive to emotionally salient stimuli, and the LPP findings suggest this sensitivity extends beyond early emotional reactivity to emotion regulation processes.

The major limitation to the present study was the ineffectiveness of the “decrease” instruction. This may have resulted from the specific wording used, or the timeframe examined of the LPP. Future research needs to examine ways to strengthen the effect of “decrease” instructional sets. Future research could also benefit from recording autonomic arousal measures concurrently with ERPs. In addition, this study did not control for menstrual phase in women, which has been shown to influence brain functioning, emotional and cognitive processes [2], [34], [35]. Finally, future research would benefit from adopting source localization and functional connectivity network analysis techniques [36] to gain greater insight into the neural networks contributing to ERP components.

These limitations notwithstanding, this study found that women have greater N1 and N2 amplitudes to negative stimuli, reflecting greater early emotional reactivity compared to men. LPP and P3 amplitudes were increased in response to “increase” instruction, and women increased LPP amplitudes to this instruction to a greater extent than men. These findings confirm predictions of the female negativity bias hypothesis and suggest that women are more sensitive to emotionally salient stimuli, and that this sensitivity extends beyond early emotional reactivity to later emotion regulation processes.

## Supporting Information

Table S1 **Stepwise Backward Regression for N100 amplitude.**
(DOCX)

Table S2 **Stepwise Backward Regression for N200 amplitude.**
(DOCX)

Table S3 **Stepwise Backward Regression for P300 amplitude.**
(DOCX)

Table S4 **Stepwise Backward Regression for LPP amplitude.**
(DOCX)
