# Supplementary material for: Sex Differences and Emotion Regulation: An Event-Related Potential Study
Source: PLoS One. 2013 Oct 30;8(10):e73475. doi: 10.1371/journal.pone.0073475 (PMC3813629; doi:10.1371/journal.pone.0073475)
Supplement: Table S1 — Stepwise Backward Regression for N100 amplitude. (DOCX) [file pone.0073475.s001.docx]

| **Predictor** | **Beta** | **t** | **Sig** |
| --- | --- | --- | --- |
| Sex | .374 | 2.698 | .010* |
| Age | .283 | 2.039 | .048* |
| **Excluded Variables** | | | |
| Depression | .161 | 1.167 | .250 |
| Anxiety | -.037 | -.256 | .799 |
| Stress | .010 | .070 | .945 |
| Reappraisal | -.022 | -.159 | .875 |
| Suppression | -.031 | -.217 | .829 |

*****p<.05
