# Supplementary material for: Sex Differences and Emotion Regulation: An Event-Related Potential Study
Source: PLoS One. 2013 Oct 30;8(10):e73475. doi: 10.1371/journal.pone.0073475 (PMC3813629; doi:10.1371/journal.pone.0073475)
Supplement: Table S2 — Stepwise Backward Regression for N200 amplitude. (DOCX) [file pone.0073475.s002.docx]

| **Predictor** | **Beta** | **t** | **Sig** |
| --- | --- | --- | --- |
| Sex | .428 | 2.975 | .005** |
| Stress | .146 | 2.184 | .035* |
| **Excluded Variables** | | | |
| Depression | .111 | .587 | .560 |
| Anxiety | .173 | 1.027 | .311 |
| Reaapraisal | -.100 | -.682 | .499 |
| Suppression | .039 | .270 | .789 |
| Age | .160 | 1.148 | .258 |

*p<.05, ** p<.005
