# Supplementary material for: Sex Differences and Emotion Regulation: An Event-Related Potential Study
Source: PLoS One. 2013 Oct 30;8(10):e73475. doi: 10.1371/journal.pone.0073475 (PMC3813629; doi:10.1371/journal.pone.0073475)
Supplement: Table S3 — Stepwise Backward Regression for P300 amplitude. (DOCX) [file pone.0073475.s003.docx]

| **Predictor** | **Beta** | **t** | **Sig** |
| --- | --- | --- | --- |
| Sex | -.347 | -2.370 | .023* |
| **Excluded Variables** | | | |
| Depression | -.054 | -.362 | .719 |
| Anxiety | .027 | .178 | .859 |
| Stress | -.040 | -.260 | .796 |
| Reappraisal | -.143 | -.971 | .338 |
| Suppression | .254 | 1.763 | .086 |
| Age | .179 | 1.225 | .228 |

*p<.05
