# Supplementary material for: Sex Differences and Emotion Regulation: An Event-Related Potential Study
Source: PLoS One. 2013 Oct 30;8(10):e73475. doi: 10.1371/journal.pone.0073475 (PMC3813629; doi:10.1371/journal.pone.0073475)
Supplement: Table S4 — Stepwise Backward Regression for LPP amplitude. (DOCX) [file pone.0073475.s004.docx]

| **Predictor** | **Beta** | **t** | **Sig** |
| --- | --- | --- | --- |
| Sex | -.361 | -2.477 | .017* |
| **Excluded Variables** | | | |
| Depression | .228 | 1.592 | .119 |
| Anxiety | .107 | .723 | .474 |
| Stress | .207 | 1.394 | .171 |
| Reappraisal | .004 | .025 | .980 |
| Suppression | -.067 | -.454 | .653 |
| Age | .165 | 1.134 | .264 |

*p<.05
